# The utility of red cell distribution width to predict mortality of septic patients in a tertiary hospital of Nepal

**DOI:** 10.1186/s12873-020-00337-8

**Published:** 2020-05-26

**Authors:** Rajan Ghimire, Yogendra Man Shakya, Tirtha Man Shrestha, Ram Prasad Neupane

**Affiliations:** Department of General Practice and Emergency Medicine, Maharajgunj Medical Campus, Institute of Medicine, Tribhuvan University, Kathmandu, Nepal

**Keywords:** Red cell distribution width, Sepsis, Emergency care, Mortality

## Abstract

**Background:**

Sepsis is a common problem encountered in the emergency room which needs to be intervened early. Predicting prognosis is always a difficult task in busy emergency rooms using present scores, which has several variables to calculate. Red cell distribution width (RDW) is an easy, cheap, and efficacious score to predict the severity and mortality of patients with sepsis.

**Methods:**

This prospective analytical study was conducted in the emergency room of Tribhuvan University Teaching Hospital among the patients age ≥ 16 years and with a clinical diagnosis of sepsis using qSOFA score. 148 patients were analyzed in the study by using a non-probability purposive sampling method.

**Results:**

RDW has fair efficacy to predict the mortality in sepsis (Area under the Curve of 0.734; 95% C. I = 0.649–0.818; *p*-value = 0.000) as APACHE II (AUC of 0.728; 95% C. I = 0.637 to 0.819; *p*-value = 0.000) or SOFA (AUC of 0.680, 95% C. I = 0.591–0.770; *p*-value = 0.001). Youden Index was maximum (37%) at RDW value 14.75, which has a sensitivity of 83% (positive likelihood ratio = 1.81) and specificity of 54% (negative likelihood ratio = 0.32). Out of 44 patients with septic shock 16 died (36.4%) and among 104 patients without septic shock, 24 died (22.9%) which had the odds ratio of 0.713 (*p* = 0.555, 95% C. I = 0.231–2.194). Overall mortality was 27.02% (*n* = 40). RDW group analysis showed no mortality in RDW < 13.1 group, 3.6% mortality in 13.1 to 14 RDW group, 22.0% mortality in 14 to > 15.6 RDW group and 45.9% mortality in > 15.6 RDW group. Significant mortality difference was seen in 14 to > 15.6 and > 15.6 RDW subgroups with a *p*-value of 0.003 and 0.008 respectively.

**Conclusion:**

Area under the curve value for RDW is fair enough to predict the mortality of patients with sepsis in the emergency room. It can be integrated with other severity scores (APACHE II or SOFA score) for better prediction of prognosis of septic patients.

## Background

Sepsis is defined as a life-threatening organ dysfunction caused by a dysregulated host response to infection. The incidence of sepsis varies among different studies with a wide range from 300 to 1000 cases/100,000 persons per year [1]. In one of the studies conducted at Tribhuvan University Teaching Hospital, 10.49% of patients showed bacterial growth in blood or bone marrow samples [2].

Organ dysfunction in the presence of infection increases in-hospital mortality by 10% [3]. One of the studies done in Nepal showed overall mortality from sepsis as 39.3% and a higher mortality rate among elderly patients (46.7%) [4]. In a comparative meta-analysis, there was 33.2% mortality of severe sepsis patients during 28-days follow up [5]. Ongoing mortality in patients with sepsis remains elevated up to 2 years and beyond [6].

Nowadays, several indicators are being used to predict the prognosis of sepsis. Commonly used prognostic indicators include Acute Physiological and chronic health evaluation II (APACHE II), Sequential Organ Failure Assessment (SOFA), Mortality in Emergency Department Score (MEDS), New York Sepsis severity score. In recent years Red cell distribution Width (RDW) is being investigated for its prognostic value in septic patients.

Red cell distribution width (RDW) is an index of variation of erythrocyte volume (i.e. anisocytosis). It is conventionally included in a standard complete blood count (CBC). The value of this parameter increases parallel with anisocytosis. It is conventionally increased in patients with anemia attributable to iron deficiency [7], folic acid/vitamin B12 deficiency, patients with autoimmune disorders [8], myelodysplastic syndrome, hemolytic anemia, liver impairment, sickle cell disease [9], and blood transfusions [10]. RDW value is increased among the red blood cell transfused patients [11] and a cutoff value of RDW to predict the mortality of critically ill patients was higher in comparison to non-transfused patients [12]. The normal range of RDW is 11.5 to 14.5% with no clinical scenarios that produce RDW < 11.5%. Any process that results in the release of reticulocytes into the circulation will increase in RDW value.

When patients are infected, microbes release various toxins/lipopolysaccharides which activate inflammatory cascade via various interleukins, cytokines [13]. Cytokines are responsible for the clinically observable effects of the bacteremia in the host [14]. These cytokines induce direct red blood cell damage by erythrophagocytosis or apoptosis, interfere with iron homeostasis, inhibit erythropoiesis by myelosuppression and downregulate erythropoietin-receptor expression [13]. These mechanisms are thought to lead to anisocytosis and increased RDW value [15].

RDW has been utilized in diverse diseases other than traditionally for the interpretation of anemia. In chronic diseases, elevated RDW was associated with all-cause mortality in critically ill patients [15, 16] and increased mortality among healthy middle-aged [17] and older adults [18] from the general population and patients with cardiovascular disease [19], stroke [20], heart failure, and chronic dialysis [21]. In acute conditions, RDW can also be used as a mortality predictor among patients with acute pancreatitis [22], subarachnoid hemorrhage [23], acute dyspnea [24] during an emergency department visit [25], out-of-hospital cardiac arrest [26], cardiac arrest in ICU [27], and critical illnesses in an ICU setting. For septic patients, RDW was also found to be an independent indicator of mortality in patients with gram-negative bacteremia, community-acquired pneumonia, severe sepsis, and septic shock [28, 29]. For every 1% increase in RDW value, total mortality risk increased by 14% among older adults [18].

In the emergency condition like sepsis, a tool that can predict the severity and thus the prognosis of a patient is crucial in deciding the modality of treatment including the vasopressor, possible need of ventilator, empiric antibiotics or higher group of antibiotics. In the resource-limited setting of developing countries like Nepal, calculating other prognostic indicators like APACHE II, MEDS, SOFA will be costly as well as time-consuming. RDW is a cost-effective and easy tool to predict the prognosis of critically ill patients including sepsis. Only a few studies of this type are conducted in developed nations and as developing nations have different health set up, this prospective analytical observational study is designed to find whether RDW can predict prognosis of septic patients in one of the tertiary centers of Nepal or not. If we can have a predicted prognosis of patients, we can decide the aggressiveness of treatment on time.

## Methods

The primary aim of this study was to determine the utility of red cell distribution width (RDW) as a prognostic factor in septic patients. The secondary aim of the study was to compare the efficacy of RDW to predict the mortality of septic patients with APACHE II and SOFA scores**.**

### Study design

This prospective observational study was conducted in Tribhuvan University Teaching Hospital (TUTH), Emergency Room, Maharajgunj, Kathmandu, Nepal from June 2017 to August 2018. Patients ≥16 years with the clinical diagnosis of sepsis in the emergency room of TUTH were included in the study. Sepsis was suspected using qSOFA (quick Sequential Organ Failure Assessment) score. Patients with infection can be predicted to have sepsis if they have at least two of following clinical criteria that together constitute a new bedside clinical score termed as quickSOFA (qSOFA): respiratory rate of 22/min or greater, altered mentation status or systolic blood pressure of 100 mmHg or less [3, 30]. Septic shock can be clinically identified by a vasopressor requirement to maintain a mean arterial pressure of 65 mmHg or greater and serum lactate greater than 2 mmol/L in the absence of hypovolemia [3]. The exclusion criteria were:

1. The patient who received blood transfusion within 90 days before emergency admission.
2. The patients who are known to have long-term conditions causing anemia like sickle cell anemia, thalassemia, iron deficiency anemia.
3. Patient with incomplete information and data.
4. The patient who deny consent.

### Sample size calculation

Sample size was 144 which was calculated using Daniel method (sample size = Z_1-α/2_^2^p(1-p)/d^2^) [31]. For this purpose,’ Z_1-α/2_^′^ is standard normal variate, 1.96 for 5% type I error; the expected proportion in population-based previous studies (p) was 10.49% [2] and ‘d’ is absolute error or precision (0.05 for 5%type I error).

### Data collection

Patients with suspected infection and hence sepsis suggested by qSOFA score were enrolled into the study after getting formal written/oral consent from the patient or legal guardian available at the Emergency room. Only septic patients meeting the inclusion and exclusion criteria were enrolled in the study without any randomization of the samples. So, it was a non-probability sampling method. Patient’s basic demographic information, vital signs on ER arrival, symptoms and underlying diseases, provisional diagnosis and laboratory values required for analysis of RDW, APACHE II, and SOFA score were collected. Clinical outcome of patients was followed by phone calls made at 28-day from the day of ER admission. Patients who were in hospital till 28-days were followed in the respective admitted wards or critical care units. Collected data were then analyzed. Data collection was done by the researcher.

### Laboratory measurements

RDW was a part of the automated complete blood count analysis. It was measured using the Nihon-Kohden automated hematology system analyzer. The normal laboratory range of RDW in our institution is 11.5 to 14.5%.

### Statistical analysis

Descriptive statistics of demographic and laboratory variables are calculated as mean, median, numbers, and percentages. Patients were further stratified a priori based on RDW values as: RDW < 13.1%; RDW ≥13.1–14%; RDW > 14–15.6%; RDW ≥15.6% [25]. An odds ratio was used to compare differences in mortality between groups. Binary logistic regression was used to evaluate potential confounding between risk factors, RDW, and mortality. Receiver operating characteristics (ROC) curve analysis was done to evaluate the performance of RDW in predicting mortality within 28-days of ER admission. The area under the ROC curve was compared between different clinical prognosis score viz. RDW, APACHE II, and SOFA. All *p*-values < 0.05 were considered statistically significant. Statistical analysis was performed using IBM SPSS (Statistical Package for Social Sciences) version 25.

## Results

A total of 148 patients were analyzed. Mean age was 51.29 years (S. D = 20.22) with a mean age in survival group 48.4 years (S. D = 19.94) and mortality group 59.10 years (S. D = 19.1). The maximum number of people lied in age-group 60–70 years (*n* = 28, 18.9%) followed by 20–30 and > 70 years both of which have the same numbers. Data is negatively skewed (− 0.217). In the study, there were more females (88, 59.5%) than males (60, 40.5%).

**Fig. 1:**
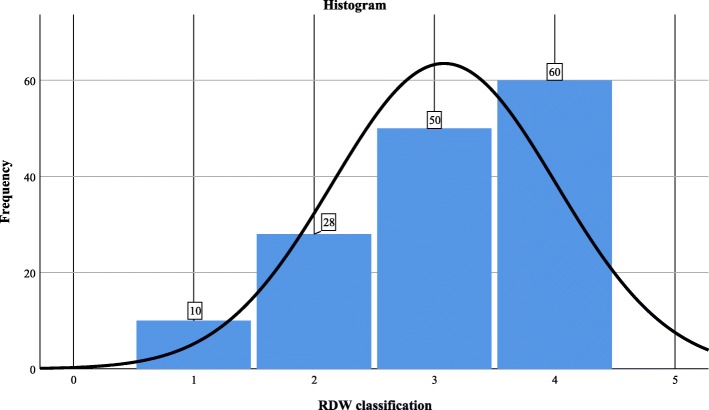
Histogram of RDW classification; 1 = RDW < 13.1, 2 = RDW ≥13.1–14, 3 = RDW > 14–15.6, 4 = RDW ≥15.6

Most of the patients lie in group with RDW > 15.6 (*n* = 60, 40.5%). (Figure 1). Mean RDW was 15.933 (S.D = 2.69). Data for RDW groups was negatively skewed (− 0.678).

As data did not follow normal distribution (negatively skewed) nonparametric test (Mann-Whitney U test) was done to test the difference of distribution of Age, RDW, APACHE II and SOFA across the categories of clinical outcome (improved and mortality). The test showed a significant difference between the improved and mortality group with a *p*-value of 0.005, 0.000, 0.000, 0.002 for age, RDW, APACHE II, SOFA respectively (Table 1).

**Table 1:** Mann-Whitney U test for predicting mortality among septic patients

Variable	Mann-Whitney U-Test	*p*-value
Age	2808.5	0.005
RDW	3422.0	0.000
APACHE II	3119.5	0.000
SOFA	2866.5	0.002

Binary logistic regression analysis was done to analyze the effect of confounding factors like age, sex, presence of septic shock on mortality. Results showed no significant effect of these confounding factors on mortality except for sex (*p* = 0.029, Odds ratio = 2.950, 95% C. I = 1.120–7.773) (Table 2). Among the predictive scores viz. RDW, APACHE II, and SOFA scores; only RDW had a significant difference in predicting mortality with an odds ratio of 1.551 (*p* = 0.000003, 95% C. I = 1.292–1.863). So RDW is a better prognostic test to predict mortality in septic patients.

**Table 2:** Binary logistic regression analysis of confounding factors and prognosis predictive scores

		Outcome						*p*-value	Odds Ratio	95% C.I	
		Improved/Cured (*N* = 108)			Mortality (*N* = 40)						
		Mean (S.D)	%	*n*	Mean (S.D)	%	*n*			Lower	Upper
Age (years)		48.4 (19.94)	73.0%	108	59.10 (19.1)	27.0%	40	0.101	1.250	0.958	1.632
Hematocrit %		35.3 (8.8)	73.0%	108	33.6 (10.1)	73.0%	40	0.315	0.979	0.941	1.020
SOFA		6 (3)	73.0%	108	8 (3)	27.0%	40	0.062	1.221	0.990	1.506
APACHE II		16 (7)	73.0%	108	21 (7)	27.0%	40	0.157	1.053	0.983	1.131
RDW		15.2 (2.2)	73.0%	108	17.9 (2.9)	27.0%	40	0.000003	1.551	1.292	1.863
Sex	Male	–	65.0%	39	–	35.0%	21	0.029	2.950	1.120	7.773
	Female	–	78.4%	69	–	21.6%	19				
Septic shock	Yes	–	63.6%	28	–	36.4%	16	0.555	0.713	0.231	2.194
	No	–	76.9%	80	–	23.1%	24				

Patients were further divided into two groups: (a) sepsis and (b) septic shock. Out of 44 patients with septic shock 16 died (36.4%) and among 104 patients without septic shock, 24 died (23.1%) with odds ratio of 0.713 (*p* = 0.555, 95% C.I = 0.231–2.194) (Table 2). Overall mortality was 27.02% (*n* = 40).

RDW group analysis showed no mortality in RDW < 13.1 group, 3.6% mortality in RDW > 13.1–14 group, 22.0% mortality in RDW > 14–15.6 group and 46.7% mortality in > 15.6) RDW group (Table 3). Significant mortality difference was seen in > 14–15.6 and > 15.6 RDW groups with *p*-value 0.003 and 0.008 respectively. This shows an increasing trend of mortality with the increase in RDW value and vice-versa.

**Table 3:** Binary logistic regression of RDW group and outcome

RDW Classification	Improved/Cured (*N* = 108)		Mortality (*N* = 40)		Odds Ratio	*p*-Value	95% C.I	
	*n*	%	*n*	%			Lower	Upper
< 13.1	10	9.3%	0	0.0%	0.000	0.003	0.000	0.000
> 13.1–14	27	25.0%	1	2.5%	0.00	0.999	0	0
> 14–15.6	39	36.1%	11	27.5%	0.042	0.003	0.005	0.332
> 15.6	32	29.6%	28	70.0%	0.332	0.008	0.139	0.746

**Fig. 2:**
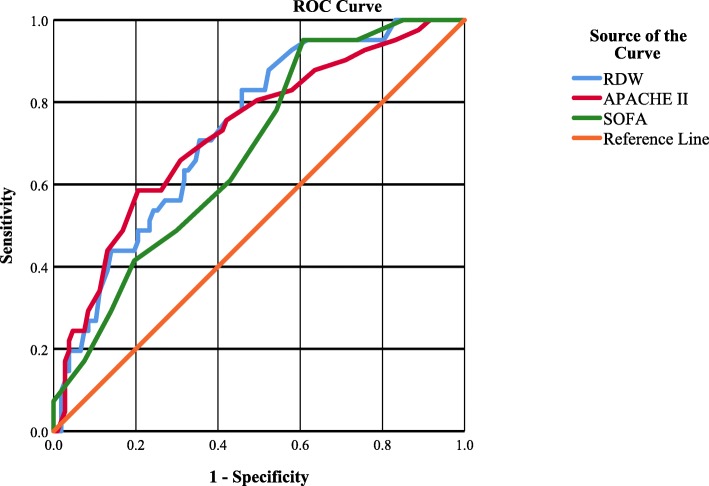
Receiver operating characteristics curve analysis for RDW, SOFA, and APACHE II to predict mortality in sepsis

Receiver Operating Characteristic (ROC) curve was used to test the efficacy of different clinical scores viz. RDW, SOFA, APACHE II to predict mortality in septic patients (Fig. 2). Area under the ROC curve was analyzed which shows RDW, APACHE II and SOFA were fair tests to predict mortality in sepsis with AUC of 0.734 (95% C. I = 0.649–0.818; *p*-value = 0.000), 0.7.28 (95% C. I = 0.637 to 0.819; p-value = 0.000), and 0.680 (95% C. I 0.591–0.770; *p*-value = 0.001) respectively (Table 4). AUC of RDW is > 0.7 which is considered a fair test.

**Table 4:** Area under the ROC curve for RDW, APACHE II, SOFA to predict mortality of sepsis

Test Variable(s)	Area	Sig.	95% Confidence Interval	
			Lower Bound	Upper Bound
SOFA	0.680	0.001	0.591	0.770
RDW	0.734	0.000	0.649	0.818
APACHE II	0.728	0.000	0.637	0.819

RDW value of 15.05 has a sensitivity of 73% (positive likelihood ratio = 1.82) and specificity of 60% (negative likelihood ratio = 0.45) while RDW value of 16.1 has sensitivity of 56% (positive likelihood ratio = 2.07) and specificity of 73% (negative likelihood ratio = 0.6). Youden Index was maximum (37%) at RDW value 14.75 which has a sensitivity of 83% (positive likelihood ratio = 1.81) and specificity of 54% (negative likelihood ratio = 0.32). Increasing the value of RDW decreases the sensitivity of the test and increases the specificity of the test.

## Discussion

This prospective analytical study illustrated the significant differences in RDW levels between mortality and survivor groups of septic patients. This study aimed to find the performance of RDW to predict the mortality of septic patients. The performance of RDW to predict mortality in septic patients was found at least equivalent to other clinical scores like SOFA, APACHE II.

Over 500,000 patients each year present to an emergency department with suspected severe sepsis [32]. Sepsis incidence increases > 100 fold with the age (0.2 per 1000 in children age 10 to 14 years to 26.2 per 1000 in those > 85 years of age) [33]. In our study, the overall mortality in septic patients was 27.02% (*n* = 40) and the mortality in septic shock patients was 36.4% which is near to mortality rate shown by a meta-analysis of multicenter randomized- trials by Stevenson EK and et al. [5] This meta-analysis had 33.2% mortality from severe sepsis.

Our study showed higher mortality in the septic shock group than patients without septic shock (36.4% vs 23.07%). In another study, among the severely septic patients (*n* = 2110), 13.8% died (*n* = 290), which is significantly higher compared with the non-severe septic group (3.8%, *n* = 187, *P* < 0.001) [25].

We found that mortality (46.7%) was more in the RDW > 15.6 group. Mortality subsequently increased with an increase in RDW value. RDW had a significant ability to predict mortality in septic patients (*p* = 0.000, Mann Whitney U Test). Kim J et al. showed that RDW was a particularly strong predictor of all-cause mortality, 30 days following critical care initiation [26].

In our study, the area under the ROC curve of RDW showed a fair capacity of RDW to predict mortality in septic patients (AUC = 0.734). AUC of RDW was greater than that of SOFA and APACHE II (AUC = 0.680 and 0.728). In another study, the area under the receiver operating characteristic curve of RDW to predict mortality was 0.75 (95% confidence interval, 0.72–0.77), which is significantly higher than the areas under the curve of clinical prediction rules (SIRS, MEDS, and CURB65) [25]. AUC of RDW is > 0.7 which is considered a fair test [34]. However, Fontana et al. showed no correlation between RDW and prognosis of septic patients [35].

Our study found that the sensitivity of RDW at 15.05 was 73% (Positive likelihood ratio = 1.82) and specificity of 60% (Negative likelihood ratio = 0.45). Decreasing the RDW value increases sensitivity while decreasing the specificity and vice versa. In a study by Chen et al.; using 12% as a cutoff of RDW, the sensitivity in predicting mortality would be 99.4% (negative likelihood ratio: 0.30). On the other hand, the specificity in predicting mortality would be 89.9% if 17% used as the cutoff of RDW (positive likelihood ratio: 3.16) [25].

There were certain limitations to our study. All the data and patients were collected in a single-center so the findings may not apply in the general population. As a purposive non-probability sampling method was used there is a chance of selection bias. The severity of the disease, patient characteristics, the value of RDW, and treatment protocol may vary with different institutes and hence the outcome of patients. Though the findings in patients with hematocrit < 36% are also applicable, patients with undiagnosed chronic anemia may have created biases and baseline hemoglobin of patients visiting the emergency room was lacking. Sepsis was diagnosed clinically using qSOFA which has low sensitivity due to which fewer cases might have been enrolled in the study.

RDW is a cheap and widely available test that has efficiency equivalent, if not more than the SOFA or APACHE II score. So it can be used in an emergency room or bedside or in a set-up where arterial blood gas analysis is not available to predict the severity/mortality of septic patients. This study provides level III evidence for its use in day by day life. However, a multicenter study involving different geographical conditions and randomized sampling method will help to reduce biases involved in the study. Separate studies need to be done before using findings to patients with anemia of different causes.

## Conclusion

RDW has fair enough efficacy to be used as a prognostic score to predict the mortality of patients with sepsis in the emergency room. RDW can be a part of the severity score along with APACHE II or SOFA score to predict mortality in septic patients. Further studies are required to confirm these data.

## Data Availability

The datasets used and/or analyzed during the current study are available from the corresponding author on reasonable request.

## Abbreviations

APACHE II: Acute Physiological and Chronic Health Evaluation II;
AUC: Area under the curve
ER: Emergency Room
qSOFA: Quick Sequential Organ Failure Assessment
RDW: Red cell distribution width
SOFA: Sequential Organ Failure Assessment
